# Isolation of a thioesterase gene from the metagenome of a mountain peak, Apharwat, in the northwestern Himalayas

**DOI:** 10.1007/s13205-012-0065-5

**Published:** 2012-06-15

**Authors:** Avneet Kour Sudan, Jyoti Vakhlu

**Affiliations:** School of Biotechnology, University of Jammu, Jammu, 180006 India

**Keywords:** Metagenomics, Multiple displacement amplification (MDA), Phi polymerase (φ), Thioesterase, α/β Hydrolase fold superfamily

## Abstract

The soil metagenome of Apharwat (latitude 34.209° and longitude 74.368°) was explored for the presence of esterase encoding genes using a cultivation-independent approach, metagenomics. Among the various protocols tested, the method developed by Wechter was found to be the best for metagenome isolation from the soil under investigation. The purity of the isolated metagenomic DNA was not suitable for gene cloning. To improve the yield and purity of isolated metagenomic DNA, isothermal amplification of the isolated metagenomic DNA using phi (φ) polymerase in a strand displacement technique was performed. The amplified DNA was comparatively pure and the yield increased 50-fold. A metagenomic library was constructed in *Escherichia coli* (DH5α) using pUC19 as a vector with an average insert size ranging between 2 and 5 kb. Out of 10,000 clones generated, one clone carrying a ~1,870-bp insert hydrolysed tributyrin, indicating esterase activity. Sequence analysis revealed that the insert harboured three open reading frames (ORFs), of which ORF 3 encoded the esterase. Open reading frame 3 comprises 1,178 bp and encodes a putative 392 amino acid protein whose size correlates with most of the bacterial esterases. The esterase isolated in the present study is suggested to be a 4-methyl-3-oxoadipyl-CoA thioesterase (Accession No. JN717164.1), as it shows 60 % sequence similarity to the thioesterase gene of *Pseudomonas reinekei* (Accession No. ACZ63623.1) by BLAST, ClustalX and ClustalW analysis.

## Introduction

Thioesterases (EC 3.1.2.23) are a large enzyme family that catalyse the hydrolysis of the thioester bond between a carbonyl group and a sulphur atom to liberate free fatty acids and thiols [coenzyme A (CoA) and acyl carrier protein]. Thioesterases are believed to have evolved from two superfamilies: (a) the α/β hydrolase fold superfamily, containing Ser-His-Asp as catalytic triad; and (b) the hotdog fold enzyme superfamily (Yokoyoma et al. 2009).

In the biosynthetic complex, two thioesterases (type I and type II) play equal roles. Type I thioesterases are responsible for removing the final product from the biosynthetic complex and type II thioesterases are reported to perform housekeeping functions, such as removing aberrant units from carrier domains. In the public databases, thioesterases are classified into 23 families, which are further characterised on the basis of sequence and three-dimensional structure similarity in the newly compiled ThYme database (Jing et al. 2011). Thioesterases have roles in the processing of secondary metabolites such as antibiotics, immunosuppressants, antitumour agents and other bioactive compounds (Ansari et al. 2004). A recent major application of thioesterases is the overproduction of biodiesel from fatty acids using microbial thioesterases (Steen et al. 2010).

To isolate novel gene sequences, including those encoding thioesterases, two main approaches are used: culture dependent and culture independent (metagenomics). The culture-dependent approach suffers from culture bias and, moreover, studies have reported that <1 % of the microbial diversity is isolated with existing culturing techniques. It is of some concern that this approach misses out >99 % of the suggested diversity. In the mid-1980s, Norman Pace (Pace et al. 1985) suggested a culture-independent approach, which was later named as “metagenomics” by Handelsman. In this technique, the isolation of environmental DNA directly, without culturing microbes, resulted in the isolation of a number of novel gene sequences (Elend et al. 2006; Vakhlu et al. 2008).

Among the commonly studied environmental samples, soil is the most widely analysed sample. It is reported that 1 g of soil contains 10 billion microorganisms. Isolation of DNA from soil is mainly affected by the presence of organic acids, such as humic acids, which interfere with enzymatic manipulations (Pang et al. 2008; Wechter et al. 2003). To improve the quality and to increase the quantity of soil DNA, the isolated DNA was amplified by multiple displacement amplification (MDA). Isolated DNA from the soil sample/s is prediluted by several fold prior to isothermal amplification using phi (φ) polymerase, which amplifies DNA by strand displacement amplification (Shoaib et al. 2008). This technique results in amplification of the DNA by several fold and the quality also increases substantially such that restriction digestion with restriction enzymes could be successfully carried out. Two approaches can be followed hereafter: (1) direct amplification of specific gene using PCR or (2) construction of a metagenomic library. To avoid the sequence bias inherent in PCR, in the present study the second approach for library construction was used.

The present study was undertaken to isolate esterase genes from the soil metagenome of Apharwat, a mountain peak in the NW Himalayas. Apharwat is located in the Baramulla District of Kashmir valley in the Jammu and Kashmir State of India, at a height of 4,267.2 m from sea level, where the temperature varies between 18 °C in summer and −20 °C in winter. The soil metagenome was selected because of its high altitude above sea level and wide temperature variation in summer and winter. This is the first report on the metagenome of soil from the northwestern Himalayas, including the isolation of a thioesterase gene with the conserved catalytic triad of Ser^137^Asp^257^His^350^.

## Methods

### Collection of soil sample

The soil sample was collected from the Apharwat Mountain (4,267.2 m) during May 2007. The soil was collected by digging 5-cm deep and collected in aseptic plastic bags that were then placed in containers to ensure that most of the microbial load was not disturbed and retained its natural form. We treated hands, trowels and ice axes with 70 % ethanol immediately before use. The samples were transported to the laboratory in dry ice, coolers and freezer packs until they were placed at −20 °C (Foght et al. 2004).

### Construction of metagenomic library

#### Isolation of metagenomic DNA

Three isolation protocols were used in the present study: Zhou’s, Pang’s and Wechter’s; however, the best results were observed using Wechter’s protocol (Zhou et al. 1996; Wechter et al. 2003; Pang et al. 2008). The DNA isolated by Wechter’s protocol contained impurities that hampered the manipulation of DNA, such as restriction digestion of the metagenomic DNA, but these were fewer in comparison with the protocols of Zhou and Pang. Although the 260/280 ratio of the metagenomic DNA isolated and pooled after following Wetcher’s protocol was ~2.3, the DNA could not be digested with various restriction enzymes to check its purity. The quality of DNA was made manipulable by carrying out an MDA reaction on the soil DNA. The standardisation method for MDA was as follows.

The 20-μl reaction assay contained DNA 1 μl (10 ng/μl), phi (φ)29 pol 0.5 μl (1 U/μl), primers 0.5 μl (100 mM/μl), IPP 1 μl (10 U/μl), DNTPs 2 μl (200 μM each) and buffer 2 μl (1×) H_2_O:13 μl. The reaction assay was started with initial denaturation at 94 °C for 3 min, followed by incubation at 30 °C for 16–17 h. After incubation, the reaction was stopped by incubation at 65 °C for 10 min. The reaction was also treated with S1 nuclease to separate debranching network from the displacement site. All the biochemicals used in the study were purchased from Genetix Asia Pvt Ltd.

### Cloning procedures

#### Partial digestion of metagenomic DNA

The metagenomic DNA was digested with *Sau*3AI to generate fragments in the range of 2–10 Kb. The enzyme incubation was standardised and the incubation range that gave the best results was determined to be 90 s. The 20-μl restriction assay comprised: metagenomic DNA; 5 μl (200 ng/μl), *Sau*3AI buffer (10×); 2 μl (1×), *Sau*3AI enzyme; 1 μl (5 U); 0.5 μl, BSA (100 mg/ml); 1 μl (100 μg/ml), Milli-Q water; 10.5 μl.

#### Ligation and transformation

Ligation of metagenomic and plasmid DNA was carried out in a ratio of 5:1 of insert:vector. The 20-μl reaction was initially placed on ice and then incubated for 16 °C overnight (16 h). The reaction comprised: metagenomic DNA; 10 μl (500 ng/μl), pUC19 DNA/*Bam*H1 digested 10 μl (100 ng/μl), T_4_ ligase; 1 μl(5 U), Buffer(10×); 2 μl (1×), Milli-Q water; 6.5 μl.

#### Transformation of *E. coli* (DH5α)

Competent cells were prepared using the TSS chemical transformation method (RoyChoudary et al. 2009). Aliquots of 60 μl of competent cells were prepared and stored at −80 °C for future use. All the chemicals used in the present study were purchased from Himedia Pvt. Ltd.

#### Transformation and screening of transformants

Transformation was carried out in *E. coli* strain DH5α. The transformation was carried out by the standard heat shock method. A total of 10,000 clones were isolated on Amp/X-gal/IPTG plates. Replica plating was performed after blue/white colonies appeared on the plates, which were then transferred onto fresh Amp/X-gal/IPTG plates incubated at 37 °C. Plasmids were isolated from the white colonies and restriction digested with *Hind*III and *EcoR*I to determine the insert size. The esterase activity of clones was checked on tributyrin plates by the agar diffusion method (Hong et al. 2007).

### Sequencing and sequence analysis using bioinformatic tools

The sequencing was done following Sanger’s dideoxy termination method (CIF, South campus, Delhi University, India). Lipase and thioesterase sequences were retrieved from NCBI and uniprot databases. Bioinformatic tools such as BLASTP for sequence alignment and ClustalW/X for multiple alignment were used. In BLASTP searches, the sequences were searched against non redundant option.

## Results and discussion

### Library construction and screening

**Fig. 1:**
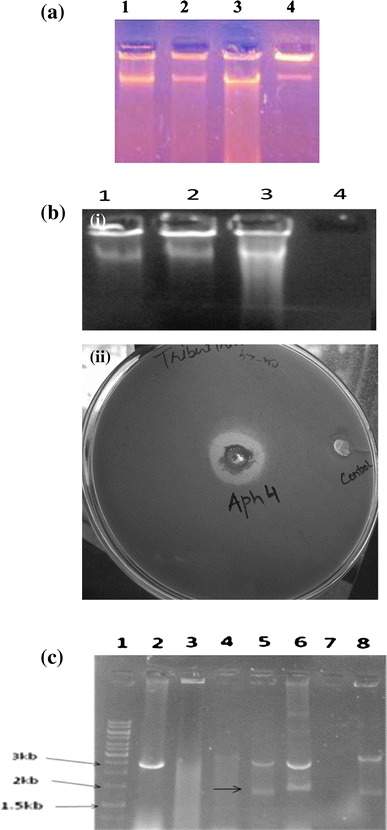
Construction of metagenomic library from Apharwat soil. **a** Standardisation of isolation of metagenomic DNA from Apharwat soil. *1* λ DNA (250 ng/μl). *2* Metagenomic DNA from Zhou’s method (100 ng/g of soil). *3* Metagenomic DNA from Pang’s method (250 ng/g of soil). *4* Metagenomic DNA from Wechter’s method (75 ng/g of soil). **b** (*i*). Multiple displacement amplification applied on Apharwat soil DNA with varying concentrations of enzymes. *Lane 1* Apharwat DNA with 0.2 U/μl of enzyme. *Lane 2* Apharwat DNA with 0.35 U/μl of enzyme. *Lane 3* Apharwat DNA with 0.5 U/μl of enzyme. *Lane 4* Apharwat DNA loaded 10 ng/μl (not visible) as control **b**. *ii* Clone Aph4 showing esterase activity on tributyrin plate. **c** Insert confirmation of the positive clone. *Lane 1* 1-Kb Marker. *Lane 2* pUC19 without insert. *Lane3*–*8* transformants digested with *Hind*III and *EcoR*1. *Arrow* depicting Aph4 insert

The Apharwat Mountain peak, situated in the northwestern Himalayas in the Indian State of Jammu and Kashmir, at a latitude 34.209° and a longitude of 74.368° (www.holyindia.org), was selected as the source of the soil metagenome. Direct DNA isolation bypasses the cultivation of the microorganisms. Therefore, contaminants from the environment adulterate the sample. In the case of the soil metagenome, the contamination is by organic acids, primarily humic acids, which share many chemical properties with DNA. The soil metagenomic DNA is usually impure and its purification is difficult because both humic acid and DNA co-precipitate with alcohol and moreover humic acid also absorbs UV at 260 nm with a λ_max_ at 230 (Sharma et al. 2007). In the present study, various isolation methods were tried. The metagenomic DNA isolation methods developed by Wechter, Zhou and Pang and their co-workers (Zhou et al. 1996; Wechter et al. 2003; Pang et al. 2008) resulted in metagenomic DNA isolation from the Apharwat soil. The yield of metagenomic DNA from Wechter’s method was 75 ng/g of soil, from Zhou’s method 100 ng/g and from Pang’s method 250 ng/g of soil (Fig. 1a). The DNA isolated by Wechter’s protocol, though at a lower concentration than that isolated by Zhou and Pang’s methods, was comparatively pure, as spectrophotometric analysis gave a 260/280 ratio of ~2.3 and agarose gel electrophoresis showed no humic acid in the background (Fig. 1a).

The metagenomic DNA isolated following Wechter’s protocol was pooled from individual isolations to construct the library in the plasmid vector. Unfortunately, the pooled DNA could not ligate into the vector pUC19. The reason for poor quality of the pooled metagenomic DNA could be that the pooling concentrates the contaminants from a low to a higher, inhibitory concentration. To increase the concentration of metagenomic DNA and reduce the concentration of the contaminants, the metagenomic DNA was diluted tenfold to 10 ng/μl. The diluted metagenomic DNA was amplified by MDA using a strand displacement technique and phi (φ) 29 to generate good quantity as well as quality metagenomic DNA (Lizardi et al. 1998; Foaster and Monahan 2005; Ballentyne et al. 2006; Shoaib et al. 2008). The amplification does not require a thermal cycler as this technique relies on the strand displacement property of phi (φ) 29 polymerase for the initiation of replication. In addition, the use of nonspecific hexanucleotide primers circumvents the bias of specific primers to large extent. The whole metagenome amplification of soil DNA from Apharwat metagenomic DNA by MDA resulted in a 50-fold increase in the concentration of the metagenomic DNA from 10 to 500 ng/μl (Fig 1b(i)). Although DNA concentrations as low as 10 pg can be amplified by MDA, in the present case, the best results were obtained with minimum of 10 ng/μl DNA and 0.5U of phi (φ) polymerase. Gonzalez et al. (2005) reported that a ten times dilution of their environmental DNA collected from the Altamira cave, which they amplified using whole genome amplification (WGA) and then applied to MDA-PCR, generated PCR products with over tenfold higher amplification as compared to standard PCR amplification (Gonzalez et al. 2005).

In another study, Neufeld et al. (2008) reported the isolation of picogram concentration of C-labelled DNA of a methanogenic microbial community. The DNA concentration was increased by MDA by several fold from an initial, very low concentration. A fosmid library was constructed that generated 10,000 clones, which when screened for methanol dehydrogenase genes, resulted in isolation of this gene (Neufeld et al. 2008).

A DNA library using amplified metagenomic DNA isolated from Apharwat soil was constructed in plasmid pUC19. The selection of pUC19 was on the basis that it has a carrying capacity of 2–8 Kb DNA and most of the microbial lipases/esterases fall in this range (Henne et al. 2000; Yun et al. 2004). A total of 10,000 white transformants were obtained with DNA inserts ranging from 2 to 5 Kb. The clones were screened on tributyrin agar plates and a clone, named Aph4, showed a clear zone formation. This indicated that the clone encoded an enzyme capable of hydrolysing tributyrin and was selected for further analysis (Fig. 1b (ii)). The size of the insert in case of Aph4 was confirmed as ~1.8 Kb by agarose gel electrophoresis (Fig. 1c).

### Sequencing

#### Sequence analysis of the open reading frames of Aph4

**Fig. 2:**
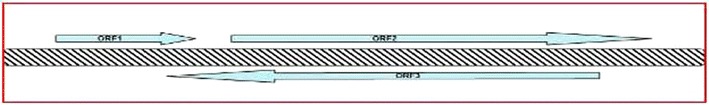
Position of ORFs located on the Aph4 sequence, drawn on the basis of ORF finder results. (1) ORF 1 395 bp, (2) ORF 2 1,286 bp, (3) ORF 3 1,178 bp

Sequencing of the insert of clone Aph4 confirmed its size as 1,870 bp. This insert sequence has a ~65 % GC content. The sequence was analysed for the presence of ORFs using the ORF finder tool of NCBI (www.ncbi.nlm.gov.orf). Three ORFs of 396 bp, 1,286 bp and 1,178 bp were found on both the strands (Fig. 2) and designated as ORF 1, ORF 2 and ORF 3. Open reading frame 1 reads from frame −2 i.e. reading from 161 to 556 bp and shows 85 % similarity to a hypothetical conserved protein of *Pseudomonas putida*. Open reading frame 2 reads from 474 to 1,760 bp on reading frame −3 and shows 54 % similarity to a protein of unknown function on BlastP analysis. Open reading frame 3 reads from 669 to 1,847 bp and its deduced protein shows 60 % similarity to a 4-methyl-3-oxoadipyl-CoA thioesterase by BlastP analysis (www.ncbi.nlm.gov.blast) (Table 1). The ORFs were non-overlapping and probably produced independent products (Table 1). The size of the esterase ORF isolated in the present study is comparable with the sizes of the ORFs of esterases reported by other studies (Table 2).

**Table 1:** List of ORF and genes of the Aph4 gene cluster

S. no.	ORF name	Length (bp)	Frame	Accession no.	Similarity with	*E* value	Similarity (%)
1	ORF 1 (131 aa)	161–556	+2	ADR61977.1	Hypothetical protein, conserved (*Pseudomonas putida*)	1e-64	87
2	ORF 2 (428 aa)	474–1,760	−3	AAG30261.1	Unknown (*Ectothiorhodospira shaposhnikomi*)	1e-45	54
3	ORF 3 (392 aa)	669–1,847	+3	ACZ63623.1	4-Methyl-3-oxoadipyl-CoA thioesterase (*Pseudomonas reinekei*)	3e-91	60

**Table 2:** List of some isolated esterase/lipase with their molecular weights

S. no.	Gene	Length of gene in terms of aminoacid composition	Molecular weight	References
1	Esterase	251 aa	27 kDa	Park et al. (2011)
2	Esterase	570 aa	63 kDa	Kang et al. (2011)
3	Esterase	507 aa	–	Tao et al. (2011)
4	Lipase	283 aa	32 kDa	Couto et al. (2010)
5	Esterase	360 aa	38 kDa	JunGang et al. (2010)
6	Esterase	–	31 kDa	Bunterngsook et al. (2010)
7	Esterases	322aa, 317 aa	–	Bayer et al. (2010)
8	Esterase	227 aa	31 kDa	Zhang et al. (2009)
9	Esterase	423 aa	44 kDa	Wu and Sun 2009
10	Esterase	–	28.6 kDa	Torres et al. (2008)
11	Esterases	380 aa	44 kDa, 260 kDa	Elend et al. (2006)
12	Esterase	392	–	This study

In a similar study Glieder et al. (2002), while isolating and expressing a 2-CL-propionic acid ester hydrolase from genomic library of *B. subtilis*, found three ORFs in the cloned fragment that showed esterase activity. The *B. subtilis* insert carried two truncated ORFs and one complete ORF that encodes an esterase in the clone. Glieder et al. (2002) cloned the esterase gene (Est4B) from *Bacillus subtilis*, which was confirmed on tributyrin plates. The protein sequence revealed high homology to the product of the *srfD* gene, a putative thioesterase gene from the surfactin synthetase gene cluster (Glieder et al. 2002). They reported that ORF 2 in the insert encodes a 243-amino acid est4B1 with 99 % sequence identity at the amino acid level to thioesterases.

**Fig. 3:**
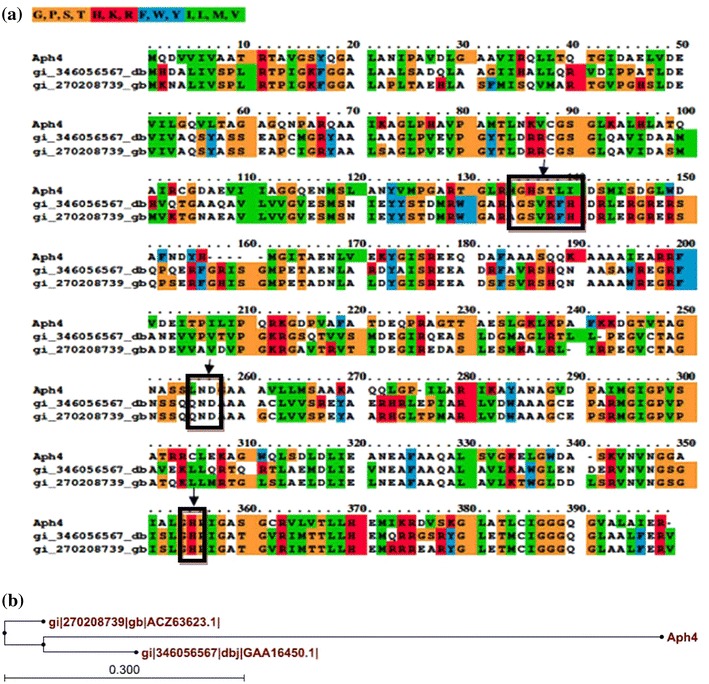
**a** Alignment between Aph4 and other known thioesterases using ClustalX.*1* ACZ63623.1 thioesterase (*P*. *reinekei*).*2* GAA16450.1 thioesterase (*P. aeruginosa*).*3* Aph4. The *arrow* depicts the catalytic triad, Ser^137^ Asp^257^ His^350^. **b** Phylogenetic tree of Aph4 and known thioesterases. The protein sequence of Aph4 and three thioesterases were aligned and the phylogenetic tree was generated using CLC Sequence Viewer software

In another study, the isolation and identification of a novel esterase from a BAC library clone with an insert size of 5.1 Kb derived from Yangtze River in China was reported (Wu and Sun 2009). Open reading frame finder analysis of that insert after sequencing indicated the presence of three ORFs, each >900 bp. One of the three ORFs was confirmed to encode an esterase (1,479–2,747 bp) on the basis of activity. The other two ORFS were a putative dehydrogenase (331–1,299 bp) and a putative penicillin binding protein (3,004–4,330 bp) (Wu and Sun 2009). In the present study, the analysis of ORF 3 was done as the clone showed activity on tributyrin plate indicating it had esterase activity. Using three software tools, i.e. BlastP, ClustalX and ClustalW, at the amino acid level indicated that ORF 3 was 60 % similar to 4-methyl-3-oxoadipyl-CoA thioesterase of *Pseudomonas reinekei* (Accession No*.* ACZ63623.1). The alignment using ClustalX software between Aph4 and selected thioesterase sequences from the public database is shown in Fig. 3a. The selected sequences for alignment were 4-methyl-3-oxoadipyl-CoA thioesterases of *P. reinekei* (ACZ63623.1) and *P. aeruginosa* (GAA16450.1). The ClustalX analysis indicated an Ser-Asp-His sequence at the position Ser^137^Asp^257^His^350^, a characteristic triad indicating that the thioesterase evolved from the α/β hydrolase fold superfamily as has been reported by Yokoyoma et al. (2009). A phylogenetic tree of Aph4 with known thioesterases showed that they evolved from the same ancestors (Fig. 3b). The phylogenetic tree was generated by CLC sequence viewer software. In addition to the presence of the triad in the sequence of Aph4, there is also a highly conserved pentapeptide sequence, G-X-S-X-L (encoded by nucleotides 1,072–1,086). Furthermore, Aph4 has conserved cysteine residues that are probably required for the formation of disulphide bonds.

**Fig. 4:**
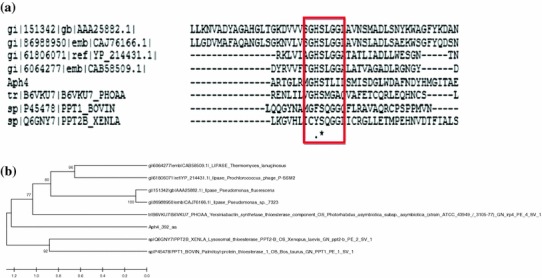
**a** ClustalW of Aph4 and known lipases having G-X-S-X-G conserved sequence. Lipase *P. Fluorescens* (gi.AAA25882.1), lipase *P. sp.*7323 (gi.CAJ76166.1), lipase *Prochlorococcus* phage P-SSM-2 (gi.Yp_214431.1), lipase *Thermomyces lanuginosus* (gi.CAB58509.1), thioesterase component of yersiniabactin synthetase gene (B6VKU7), lysosomal thioesterase (Q6GNY7) and palmitoyl protein thioesterase (P45478). **b** Phylogenetic tree of Aph4, known lipases and known thioesterase that have same GXSXG motif generated by using Mega5 software

Conserved pentapeptide sequence that is found in known lipases and esterases, including thioesterase at their active site, is G-X-S-X-G (Brumlik and Buckley. 1996; Kim et al. 2006), whereas in thioesterase under study the conserved site is GHSTL. Sequences of known lipases and thioesterase with the GXSXG conserved motif were retrieved from the NCBI and Uniprot databases for comparison in the present study. The selected lipase/esterase sequences are *Pseudomonas fluorescens* (gi.AAA25882.1), *P. sp.*7323 (gi.CAJ76166.1), lipase *Prochlorococcus* phage P-SSM-2 (gi.Yp_214431.1), *Thermomyces lanuginosus* (gi. CAB58509.1), yersiniabactin thioesterase PHOAA (B6VKU7), palmitoyl protein thioesterase 1(P454782) and lysosomal thioesterase (Q6GNY7). Multiple alignments with ClustalW software, of known lipases and thioesterases (taken from uniprot) with Aph4 indicated that the nucleophilic serine in the active site was conserved in thioesterase under study as well (Fig. 4a). However, the last conserved residue glycine was replaced with leucine in the present case. There are reports concerning the presence of G-X-S-X- instead of G-X-S-X- , in which the active serine is located in the middle of the sequence GXSXS (Brumlik and Buckley 1996). In Aph4, the serine was found to be located in the middle of the sequence GHSTL. This conserved region is reported in lipases and esterases including thioesterase (Kim et al. 2006).

Liaw et al. (2010) analysed 12 isolated lipolytic genes using different bioinformatic tools, such as ORF finder and BlastP. They reported the presence of 16 ORFs that encoded proteins showing similarity to proteins with lipolytic activity. Among these ORFs, ten possessed the conserved pentapeptide GXSXG motif, two ORFs had the GDSL motif, three ORFs showed a GDSAG motif, two ORFs possessed a GFSQG motif, and one ORF carried an AXSXG motif instead of GXSXG, which showed lipolytic activity towards tributyrin plates (Liaw et al. 2010). These results indicate that although the GXSXG motif is the most conserved in lipolytic proteins including thioesterases, other conserved catalytic motifs have also evolved.

A phylogenetic tree using mega 5 software was used to determine the active site serine position between Aph4 and four lipase and three thioesterases sequences as they share common conserved pentapeptide GXSXG sequence (Fig. 4b). The phylogenetic tree revealed that this thioesterase has diverged during evolution and that the distance between it and the known thioesterases is reasonable. Thus, Aph4 can be termed as a novel esterase.

Liebeton et al. (2001) reported that two cysteines form a disulphide bond, which are required for stabilisation of the lipase structure. Other reports indicate that cysteine and histidine are conserved in thioesterases. Ye et al. (1999) isolated a thioesterase gene whose encoded protein did not have the conserved pentapeptide sequence, but was rich in cysteine and histidine sequences. Cho and Cronan (1993) have also reported the presence of histidine in the highly conserved motif G-X-H near the carboxyl terminal of thioesterase enzymes isolated from *E. coli*.

The sequence comparisons confirm ORF 3 in the clone APH4 carrying insert from the Apharwat soil metagenome and encoding an esterase, which is a thioesterase. The sequence of ORF 3 has been submitted to NCBI under the accession number JN717164.1.

## Conclusion

No single method of metagenomic DNA isolation can suffice for all soil types. Therefore, various available methods and their modified versions need to be tested to isolate good quality metagenomic DNA for the specific soil. If the yield of metagenomic DNA is low, DNA isolated from various isolations is routinely pooled. This pooling also results in the pooling of humic acid and other organic acids, which are inhibitors of the various enzymes used in gene cloning. To circumvent this problem, the DNA is diluted (thus, the contaminants are also diluted) and the whole metagenome is amplified using phi polymerase (φ) by strand displacement amplification. We conclude that instead of pooling the metagenomic DNA isolated from different isolations, a single DNA isolation can be diluted and can serve as the template for whole genome amplification. Genes can be isolated from enriched metagenomes directly by PCR; however, PCR is biased towards already known sequences and novel genes may be missed. Instead of using PCR, a metagenomic library was constructed and clones were screened for esterase by functional screening using tributyrin agar plates. The activity was measured by the zone of hydrolysis, observed on tributyrin plates. The phylogenetic tree of related thioesterases, lipases and Aph4 indicated that Aph4 was indeed a thioesterase gene.
